# Pediatrics Residents’ Conference Attendance Does Not Predict Their In-Training Exam Scores

**DOI:** 10.7759/cureus.87072

**Published:** 2025-06-30

**Authors:** Adin Nelson, Anjile An, Matthew Kapklein, Erika Abramson

**Affiliations:** 1 Pediatrics, Weill Cornell Medicine, New York, USA; 2 Population Health Sciences, Weill Cornell Medicine, New York, USA; 3 Pediatrics, Westchester Medical Center, Valhalla, USA

**Keywords:** curriculum, didactics, education, exam preparation, residency

## Abstract

Objective

Residents work hard both at their clinical duties and at their learning, and they have limited time in which to balance the two. Existing evidence on the value of didactic conferences is mixed, and prior studies predate the increases in virtual and asynchronous learning since COVID-19. To address that gap, we explored the relationship between residents’ attendance at didactic conferences and their medical knowledge as measured on annual standardized exams.

Methods

We conducted a retrospective national study assessing the relationships between pediatrics residents’ attendance at didactic conferences and their scores on the American Board of Pediatrics In-Training Exam (ITE) from July 2022 through July 2023, adjusting for residents’ level of training and prior exam scores.

Results

We analyzed data from 383 residents from 10 programs, and we found no significant correlation between conference attendance and ITE scores.

Conclusions

Didactic sessions are a required component of residency training, but we found that attendance at these sessions does not correlate with residents’ performance on standardized exams. This highlights the need for future research exploring how didactic sessions can be made more effective and whether conference attendance might impact other outcomes such as clinical skills more than exam performance.

## Introduction

Residents face myriad demands on their precious time. Patient care, self-care, administrative tasks, clinical learning, and didactic educational sessions can easily take up more hours than there are in a week. All of those activities are essential, so limited time leads to perennial debates over which are the most essential and how to prioritize them. 

Evolutions in medical education since the COVID-19 pandemic have exacerbated that tension. During the pandemic, many universities limited or canceled in-person learning in favor of asynchronous online learning [1-3]. That produced a cohort of trainees who are accustomed to learning at their own pace in their own time and, conversely, unaccustomed to in-person lectures and workshops [4,5]. At the same time, there has been a rapid proliferation of medical education podcasts, videos, and exam preparation materials that medical students and residents increasingly use as their primary learning resources [2,6]. That raises renewed questions about the value of traditional, synchronous, in-person, didactic sessions such as morning report and noon conference.

A number of previous studies have explored the educational value of residents’ attendance at didactic conferences [7-16], but their results have been mixed. Additionally, most prior studies were small or single-center studies, and they predate the COVID-19 pandemic, so they are difficult to generalize broadly or apply to current circumstances.

We aimed to fill those literature gaps by conducting a national study examining the relationship between residents’ attendance at scheduled didactic sessions (e.g. morning report, noon conference, academic half day, etc.) and their medical knowledge as measured on national standardized exams. Understanding the value of traditional conferences can help educators optimize their programs to protect residents’ learning time and maximize the quality of didactic sessions.

## Materials and methods

We adopted a post-positivist orientation and conducted a retrospective observational study of the relationship between residents’ attendance at regularly scheduled didactic conferences and their medical knowledge as measured on annual standardized tests. We reached out to pediatrics residency program directors through the Association of Pediatric Program Directors (APPD) email list and invited them to contribute data about their first-year (postgraduate year (PGY)-1) and second-year (PGY-2) residents from academic year 2022-2023.

Participating programs submitted a de-identified spreadsheet listing each resident’s level of training in academic year 2022-2023 (PGY-1 vs PGY-2), the percentage of assigned didactic conferences they attended that year, and their scores on the 2022 and 2023 American Board of Pediatrics In-Training Exams (ITE). Programs reported both residents’ overall conference attendance and their attendance at specific conference series such as morning report, noon conference, grand rounds, and academic half days. We analyzed those individual conference types and also compared daily noon conference vs weekly academic half-day schedules. The leadership of each program recorded residents' attendance according to their own policies. Programs reported residents’ attendance as the percentage of conferences they attended out of those that they were assigned or expected to attend, accounting for their clinical schedule. That calculation makes it possible for residents’ attendance to be >100% if they attended sessions that they were not expected to attend.

We chose the pediatrics ITE as our outcome metric because it is administered at the very beginning of the academic year, so it aligns well with exposures such as conference attendance that fall within a single academic year. ITE scores have previously been shown to significantly predict board certification results [17], and board exam performance has been shown to predict new physicians’ patients’ clinical outcomes [18], so there is good validity evidence for using ITE scores in research.

We included all data submitted by participating programs. We excluded residents who had incomplete data because they did not take the ITE in 2022 and/or 2023 or had missing attendance records.

We used linear regression models to evaluate the relationships between residents’ conference attendance, level of training, 2022 and 2023 ITE scores, and 2022 to 2023 change in ITE scores. We used the Welch two-sample t-test and Fisher’s exact test to compare the mean and standard deviation of these characteristics between PGY-1 and PGY-2 residents. We also checked for significant differences between participating programs using Fisher’s exact test, Kruskal-Wallis rank-sum test, and multivariable linear regression. All analyses were performed in R Version 4.4.1 (R Foundation for Statistical Computing, Vienna, Austria), and we evaluated significance at an alpha of 0.05. The Weill Cornell Medicine Institutional Review Board deemed this study non-human subjects research (IRB record number: 24-04027273-01).

## Results

We received data on 559 residents from 10 residency programs in the 2022-2023 academic year. We excluded 175 residents because we did not have complete data; 141 were PGY-3 residents who graduated in June 2023 and therefore could not take the July 2023 ITE; 33 PGY-1 or PGY-2 residents were missing 2022 and/or 2023 ITE scores; and one resident was missing attendance data. Residents with incomplete data were randomly distributed throughout all participating programs.

We included 383 residents in our analysis. Participating programs represent all regions of the U.S. and a broad mix of small, medium, and large programs. Names and locations of participating programs are in Table 1. De-identified data about the individual programs appear in Table 2. There are significant differences among the programs in 2022 ITE score, 2023 ITE score, ITE score changes, and overall conference attendance. There is no significant relationship between program size and 2022 ITE scores (p = 0.081), but there is a significant positive association between program size and ITE change (β = 0.13, 95% CI: 0.07, 0.17, p < 0.001). There is also a significant difference in residents’ conference attendance between programs using noon conferences and programs using academic half days (average attendance 45.5% vs 100.1%, respectively, p < 0.001), but no significant difference in average ITE score change (10.6 vs 11.4 points, p = 0.69).

**Table 1:** Pediatrics residency programs that contributed data to this study. The order of programs in this table is alphabetical; it does not correlate to the program ID number assigned in the study.

Program	Location
Children's Hospital of Los Angeles	Los Angeles, CA
East Tennessee State University	Johnson City, TN
George Washington University School of Medicine and Health Sciences	Washington, DC
SUNY Upstate	Syracuse, NY
UCSF Benioff Children's Hospital	Oakland, CA
University of Florida	Pensacola, FL
University of Louisville	Louisville, KY
Valley Children’s Healthcare	Madera, CA
Wake Forest	Winston-Salem, NC
Weill Cornell Medicine	New York, NY

**Table 2:** Summary data about the residency programs included in this study. AHD indicates weekly Academic Half Day didactic sessions; NC indicates daily Noon Conference sessions. Program-level analyses are linear regressions of overall attendance vs In-Training Exam (ITE) score change. Program comparisons are the results of Kruskal-Wallis tests. *Indicates comparisons that are statistically significant at an alpha of <0.05. Note that program identifiers in this table do not correlate with the order in which the programs are listed in Table 1.

Program	Size (total residents)	Schedule	2022 ITE	2023 ITE	ITE Change	Overall Attendance (%)	Program-level regression analysis		
							β	95% CI	p
P01	42	AHD	155	168	12	107	0.03	-0.23, 0.29	0.87
P02	27	NC	144	153	10	94	-0.42	-2.8, 2.0	0.46
P03	111	NC	154	164	10	43	0.31	-0.12, 0.73	0.1
P04	45	AHD	153	159	6	99	-0.33	-2.5, 1.9	0.73
P05	62	NC	149	156	7	67	0.13	-0.22, 0.48	0.75
P06	24	NC	143	148	4	69	0.45	-0.31, 1.2	0.64
P07	39	AHD	141	157	16	93	-0.93	-2.2, 0.34	0.36
P08	50	NC	148	155	6	41	0.02	-0.36, 0.39	0.7
P09	98	NC	148	158	10	23	1	0.28, 1.8	0.049*
P10	120	NC	151	169	18	38	0.13	-0.03, 0.29	0.42
Comparison:	-	-	p = 0.23	p < 0.001*	p < 0.001*	p < 0.001*	-	-	-

Across all programs, PGY-2 residents scored significantly higher than PGY-1 residents on both the 2022 and 2023 ITE, and 2023 ITE scores were significantly higher than 2022 scores for both PGY-1 and PGY-2 residents (p < 0.001 for both comparisons, see data in Table 3). For context, national average ITE scores for 2022 and 2023 are also in Table 3, and study participants’ scores are not notably different from the national averages. 

**Table 3:** Study participants’ and national mean PGY-1 and PGY-2 In-Training Exam (ITE) scores from 2022 and 2023. Note that the postgraduate year (PGY) level labeled in this table refers to the residents’ year in training during the 2022-2023 academic year, so the scores labeled “PGY-1 2023” are actually those residents’ PGY-2 scores, and the scores labeled “PGY-2 2023” are actually those residents’ PGY-3 scores. (Superscripts a, b, c, and d indicate statistically-significant differences by the Welch two-sample t-test; p < 0.001 in all cases.)

	Study PGY-1 [mean (SD)]	National PGY-1 [mean (SD)]	Study PGY-2 [mean (SD)]	National PGY-2 [mean (SD)]
2022	143^a,c^ (17)	143 (18)	157^a,d^ (16)	158 (17)
2023	157^b,c^ (20)	156 (19)	164^b,d^ (16)	164 (20)

In multivariable linear regression analysis of the individual programs, we found no significant correlation between residents’ conference attendance and their 2022-2023 ITE score change in nine of 10 programs; only one program showed a significant positive correlation (P09: β = 1.0, 95% CI: 0.28, 1.8, p = 0.049).

Analyzing the pooled data, all residents’ 2022 ITE scores positively and significantly predicted their 2023 ITE scores (β = 0.7, 95% CI: 0.63, 0.77, p < 0.001), but their overall conference attendance did not predict their subsequent scores (β = 0.00, 95% CI: -0.05, 0.04, p = 0.81) or the change in their ITE scores (PGY-1: β = 0.00, 95% CI: -0.07, 0.07, p = 0.99; PGY-2: β = -0.01, 95% CI: -0.08, 0.05, p = 0.66). There was no significant relationship between PGY level and overall attendance (β = -0.03, 95% CI: -0.12, 0.06, p = 0.47), and individual regressions analyzing PGY-1 and PGY-2 residents separately produced the same results. We tested for relationships between ITE scores and residents’ attendance at particular types of conferences (e.g. morning report, noon conference, grand rounds, academic half day), and we again found no significant correlations. When we included program size and conference attendance in the same analysis, though, overall attendance does significantly predict ITE score changes: β = 0.11, 95% CI: 0.04, 0.17, p = 0.001.

## Discussion

In this large national study, we found no significant correlation between pediatrics residents’ attendance at didactic conferences and their ITE scores at nine of 10 individual programs and in the pooled data. Didactic sessions are mandated by the Accreditation Council for Graduate Medical Education (ACGME), and they are considered an essential component of residency training, yet our results suggest that attending these sessions does not correlate with growth in residents' medical knowledge, at least as measured on the pediatric ITE.

Previous smaller studies in this area have found mixed results. Studies in medicine [7,14], family medicine [12,13], and emergency medicine [8] have similarly shown no correlation between didactic conference attendance and knowledge gains, but other studies in both medicine [9,10] and surgery [15,16] have found significant positive correlations between conference attendance and annual exam scores. It can be challenging to apply evidence from one field of medicine to another, but this existing literature spans the full range from primarily cognitive to primarily procedural disciplines, and there is no evidence to suggest that the value of didactic conferences varies by specialty. There may be program-specific factors such as conference curriculum structure, conference style, or ITE score requirements for promotion that influence the relationship between conference attendance and ITE scores, but our study was not designed to assess those details. 

One interesting study in a medicine residency program in Thailand [11] found that different types of didactic conferences had different effects; attendance at lectures did not correlate with either residents’ medical knowledge or their clinical skills, but attendance at non-lecture workshops did significantly correlate with improvements in residents’ clinical skills. That dichotomy raises an important point: most studies in this area have focused on residents’ medical knowledge as assessed on written exams, and exam performance may not correlate perfectly with clinical performance. A recent study did show that physicians’ performance on the American Board of Internal Medicine certification exam did predict their patients’ clinical outcomes in their first years of practice though [18], so concerns about the disconnect between exam performance and clinical performance may be overstated. 

The question of different effects of different types of didactic sessions remains, and our study was not designed to answer it. In collecting data from 10 residency programs, we accounted for different conference types as described by the individual programs but we were unable to differentiate lecture-based from interactive skills-based sessions. If attendance at some, but not all, didactic sessions does contribute to residents’ medical knowledge, then grouping all conferences together may have masked that effect in our results. Similarly, we only assessed residents’ performance on written exams; if attendance at some - or all - didactic sessions contributed to clinical skills, our study could not measure that impact.

Another limitation to our study is that each program collected its own attendance data. All programs reported the percentage of conference sessions a resident attended out of those they were expected to attend, but there may have been differences in how programs measured attendance or how they determined which conferences a resident was supposed to attend.

One interesting and unexpected result from our data is the significant positive correlation between program size and average ITE score changes, and the fact that after accounting for program size, conference attendance does significantly predict ITE score changes. There is no immediately intuitive reason why a larger program should have a more effective didactic curriculum, unless number of residents in a program is a proxy for number of faculty, program resources, or other factors. Future studies should explore that further.

Our study suggests two important next steps for this research: First, future studies should gather specific information about the content, style, and quality of didactic conferences to test whether more-interactive sessions or higher-rated sessions contribute more to residents’ learning. Second, future studies should include broader outcomes to test the possibility that conference attendance may contribute to clinical skills more than it does to medical knowledge as assessed on exams. Next steps in this research should also include assessing residents’ milestones or Entrustable Professional Activities (EPA) ratings as well as exam scores.

## Conclusions

Didactic conference sessions are a mandatory and essential part of residency training, yet multiple small studies in multiple fields have shown mixed results as to their efficacy. The existing evidence spans both medical and surgical specialties in a variety of settings. In this large national study of pediatrics residents, we found no significant overall correlation between residents’ conference attendance and their medical knowledge as measured on annual standardized exams. That raises challenging questions about the utility of this traditional mode of teaching, and it demands rigorous further research.

## References

[1] Stojan J, Haas M, Thammasitboon S (2022). Online learning developments in undergraduate medical education in response to the COVID-19 pandemic: a BEME systematic review: BEME Guide No. 69. Med Teach.

[2] Binks AP, LeClair RJ, Willey JM (2021). Changing medical education, overnight: the curricular response to COVID-19 of nine medical schools. Teach Learn Med.

[3] Jeffries PR, Bushardt RL, DuBose-Morris R (2022). The role of technology in health professions education during the COVID-19 pandemic. Acad Med.

[4] Sodeify R, Habibpour Z, Akbarbegloo M (2022). Explaining medical students' perceptions of asynchronous virtual education in the COVID-19 pandemic: a qualitative study. J Educ Health Promot.

[5] Chang MF, Yeh CC, Lue JH, Liao ML (2024). Medical students' preferences for asynchronous online or face-to-face learning strategies in learning gross anatomy and neuroanatomy during the COVID-19 pandemic. Clin Anat.

[6] Delungahawatta T, Dunne SS, Hyde S, Halpenny L, McGrath D, O'Regan A, Dunne CP (2022). Advances in e-learning in undergraduate clinical medicine: a systematic review. BMC Med Educ.

[7] Cacamese SM, Eubank KJ, Hebert RS, Wright SM (2004). Conference attendance and performance on the in-training examination in internal medicine. Med Teach.

[8] Gene Hern H Jr, Wills C, Alter H, Bowman SH, Katz E, Shayne P, Vahidnia F (2009). Conference attendance does not correlate with emergency medicine residency in-training examination scores. Acad Emerg Med.

[9] McDonald FS, Zeger SL, Kolars JC (2008). Associations of conference attendance with internal medicine in-training examination scores. Mayo Clin Proc.

[10] McDonald FS, Zeger SL, Kolars JC (2007). Factors associated with medical knowledge acquisition during internal medicine residency. J Gen Intern Med.

[11] Limvorapitak W (2016). Correlation of academic activity attendance and examination scores of internal medicine residents. J Med Assoc Thai.

[12] Picciano A, Winter R, Ballan D, Birnberg B, Jacks M, Laing E (2003). Resident acquisition of knowledge during a noontime conference series. Fam Med.

[13] Winter RO, Picciano A, Birnberg B (2007). Resident knowledge acquisition during a block conference series. Fam Med.

[14] FitzGerald JD, Wenger NS (2003). Didactic teaching conferences for IM residents: who attends, and is attendance related to medical certifying examination scores?. Acad Med.

[15] Godellas CV, Hauge LS, Huang R (2000). Factors affecting improvement on the American Board of Surgery In-Training Exam (ABSITE). J Surg Res.

[16] Godellas CV, Huang R (2001). Factors affecting performance on the American Board of Surgery in-training examination. Am J Surg.

[17] Althouse LA, McGuinness GA (2008). The in-training examination: an analysis of its predictive value on performance on the general pediatrics certification examination. J Pediatr.

[18] Gray BM, Vandergrift JL, Stevens JP, Lipner RS, McDonald FS, Landon BE (2024). Associations of internal medicine residency milestone ratings and certification examination scores with patient outcomes. JAMA.

